# Complete Genome Sequence of an Ebola Virus Isolate Imported from Sierra Leone to Germany Determined by Circle Sequencing 

**DOI:** 10.1128/genomeA.01011-16

**Published:** 2016-10-06

**Authors:** Jan Philipp Mengel, Artur Lissin, Nadine Biedenkopf, Tilman Schultze, Gopala Krishna Mannala, Gordian Schudt, Gerrit Kann, Timo Wolf, Markus Eickmann, Stephan Becker, Franz Cemic, Torsten Hain

**Affiliations:** a Institute for Medical Microbiology, Justus-Liebig University of Giessen, Giessen, Germany; b Institute for Biochemical Engineering and Analytics, University of Applied Sciences, Giessen, Germany; c Institute of Virology, Philipps University of Marburg, Marburg, Germany; d German Center for Infection Research (DZIF), partner site Giessen-Marburg-Langen, Germany; e Department of Medicine, Infectious Diseases Unit, University Hospital Frankfurt, Frankfurt am Main, Germany

## Abstract

We report here a complete genome sequence of Ebola virus Makona from a nonfatal patient sample that originated in Sierra Leone during the last Ebola virus outbreak in West Africa (species *Zaire ebolavirus*) using a highly accurate circle sequencing (Cir-seq) method.

## GENOME ANNOUNCEMENT

Ebola virus causes a severe fever with high fatality rates in humans (1). The recent Ebola outbreak in West Africa during 2013 to 2016, affecting mainly Guinea, Liberia, and Sierra Leone, was the largest ever recorded (28,646 cases and 11,323 fatalities) (2). Here, we report the complete genome sequence of a virus isolate from a patient who contracted the disease in Sierra Leone and was treated in Frankfurt, Germany (3). The virus was isolated from a highly positive serum sample (day 9) and passaged two times each in Vero E6 and HuH7 cells for virus propagation (sequences from p4 stocks). RNA was extracted from cell supernatants using the Qiagen RNeasy kit, according to the manufacturer’s instructions.

We used a novel circle sequencing approach (4), a technology that allows highly accurate genome sequencing of RNA virus genomes, with optimization as described below. Prior to RNA fragmentation, the sample was depleted using Ribo-Zero Gold kit (Illumina), as recommended by the manufacturer. The resulting rRNA-free sample was fragmented with ZnCl_2_ for 11 min at 94°C (Ambion). After, it was purified using a 15% Tris-borate-EDTA (TBE)-urea gel (Novex) and stained with SYBR Gold (Life Technologies). RNA fragments corresponding to 120 to 190 nucleotides (nt) in length were cut and crushed using gel breaker tubes (IST Engineering, Inc.). The crushed gel was eluted overnight in 360 µl of elution buffer (0.6 M sodium acetate, 0.01% SDS, 0.001% EDTA). Gel debris was removed afterward with a spin filter, and purified RNA was precipitated with 100% ethanol after the addition of glycogen (Illumina). After centrifugation (21,000 × *g*), the pellet was washed with 70% ethanol, dried, and suspended in nuclease-free water. Purified fragments were 5′-phosphorylated circularized with T4 polynucleotide kinase (NEB) and T4 RNA ligase (NEB) at 37°C and extracted with phenol:chloroform:isoamyl alcohol (Roth), followed by ethanol precipitation. Circularized RNA templates were then reverse transcribed with 400 U of SuperScript III (Invitrogen), and RNA templates were digested with 8 mU of RNase H (Invitrogen). The following steps of second-strand synthesis, 3′-adenylation, sequencing adapter ligation, and PCR amplification (15 cycles) were performed according to Illumina’s TruSeq Total RNA protocol. After, the sample was purified on a 6% TBE gel (Novex), and the fragments sized from 450 to 600 nt were cut and eluted in 10 mM Tris-EDTA (TE) buffer. Finally, the DNA was precipitated in 100% ethanol, washed in 70% ethanol, and suspended in nuclease-free water. The library was sequenced on an Illumina MiSeq using version 3 chemistry and 2 × 300 cycles.

Circle sequencing (Cir-seq) raw read output was preprocessed as described previously (4). Second, the preprocessed reads were aligned to the complete genome of *Zaire ebolavirus* isolate (accession no. KJ660347.2) using Bowtie 2 (version 2.2.6) (5), yielding an average coverage of approximately 5,200-fold. Subsequently, bases with a Phred score lower than 20 were discarded, and for each reference position, the base with the highest relative frequency was selected as the consensus base. The minimal relative frequency of a consensus base was thus determined to be 91.5%.

### Accession number(s).

The complete genome sequence of the *Zaire ebolavirus* isolate Ebola virus H.sapiens-tc/SLE/2014/Makona-Frankfurt has been deposited at the European Nucleotide Archive under the accession no. LT605058.
